# Efficacy of surgical skin preparation with chlorhexidine in alcohol according to the concentration required to prevent surgical site infection: meta-analysis

**DOI:** 10.1093/bjsopen/zrac111

**Published:** 2022-09-19

**Authors:** Tatsuki Hasegawa, Sho Tashiro, Takayuki Mihara, Junya Kon, Kazuki Sakurai, Yoko Tanaka, Takumi Morita, Yuki Enoki, Kazuaki Taguchi, Kazuaki Matsumoto, Kazuhiko Nakajima, Yoshio Takesue

**Affiliations:** Division of Pharmacodynamics, Keio University Faculty of Pharmacy, Minato-ku, Tokyo, Japan; Division of Pharmacodynamics, Keio University Faculty of Pharmacy, Minato-ku, Tokyo, Japan; Division of Pharmacodynamics, Keio University Faculty of Pharmacy, Minato-ku, Tokyo, Japan; Division of Pharmacodynamics, Keio University Faculty of Pharmacy, Minato-ku, Tokyo, Japan; Division of Pharmacodynamics, Keio University Faculty of Pharmacy, Minato-ku, Tokyo, Japan; Division of Pharmacodynamics, Keio University Faculty of Pharmacy, Minato-ku, Tokyo, Japan; Division of Pharmacodynamics, Keio University Faculty of Pharmacy, Minato-ku, Tokyo, Japan; Division of Pharmacodynamics, Keio University Faculty of Pharmacy, Minato-ku, Tokyo, Japan; Division of Pharmacodynamics, Keio University Faculty of Pharmacy, Minato-ku, Tokyo, Japan; Division of Pharmacodynamics, Keio University Faculty of Pharmacy, Minato-ku, Tokyo, Japan; Department of Infection Prevention and Control, Hyogo College of Medicine, Nishinomiya, Hyogo, Japan; Department of Infection Prevention and Control, Hyogo College of Medicine, Nishinomiya, Hyogo, Japan; Department of Clinical Infectious Diseases, Tokoname City Hospital, Tokoname, Aichi, Japan

## Abstract

**Background:**

A combination of chlorhexidine gluconate and alcohol (CHG–alcohol) is recommended for surgical skin preparation to prevent surgical site infection (SSI). Although more than 1 per cent CHG–alcohol is recommended to prevent catheter-related bloodstream infections, there is no consensus regarding the concentration of the CHG compound for the prevention of SSI.

**Methods:**

A systematic review and meta-analysis was performed. Four electronic databases were searched on 5 November 2020. SSI rates were compared between CHG–alcohol and povidone-iodine (PVP-I) according to the concentration of CHG (0.5 per cent, 2.0 per cent, 2.5 per cent, and 4.0 per cent).

**Results:**

In total, 106 of 2716 screened articles were retrieved for full-text review. The risk ratios (RRs) of SSI for 0.5 per cent (6 studies) and 2.0 per cent (4 studies) CHG–alcohol were significantly lower than those for PVP-I (RR = 0.71, 95 per cent confidence interval (c.i.) 0.52 to 0.97; RR = 0.52, 95 per cent c.i 0.31 to 0.86 respectively); however, no significant difference was observed in the compounds with a CHG concentration of more than 2.0 per cent.

**Conclusions:**

This meta-analysis is the first study that clarifies the usefulness of an alcohol-based CHG solution with a 0.5 per cent or higher CHG concentration for surgical skin preparation to prevent SSI.

## Teaser

The risk ratios (RRs) of SSI for 0.5 per cent and 2.0 per cent chlorhexidine gluconate (CHG)-alcohol were significantly lower than those for povidone-iodine (RR = 0.71, 95 per cent c.i. 0.52 to 0.97; RR = 0.52, 95 per cent c.i. 0.31 to 0.86 respectively). We first clarified the usefulness of an alcohol-based CHG solution with a 0.5 per cent or higher CHG concentration for surgical skin preparation to prevent surgical site infection.

## Introduction

Surgical site infection (SSI) is the third most common category of healthcare-associated infections, with a prevalence of 15.7 to 31.0 per cent among all healthcare-associated infections^1,2^. In one systematic review, most studies revealed an economic benefit associated with prevention of SSI^3^. Several guidelines for the prevention of SSI have been published^4–6^. Approximately half of SSIs are estimated to be preventable by application of evidence-based strategies^7^. A bundle approach is suggested to decrease SSIs, and surgical site skin preparation is one of the essential elements used in bundle approaches^8–10^. In particular, Leaper and Ousey^11^recommended the use of a 2 per cent chlorhexidine gluconate in alcohol (CHG–alcohol) skin preparation, postoperative negative-pressure wound therapy, and antiseptic surgical dressings because of the high quality of evidence. Two meta-analyses suggested a significant benefit of using CHG–alcohol compared with aqueous povidone-iodine (PVP-I) with moderate-quality evidence^6,12^; however, a low quality of evidence was shown when the risk of SSI was compared between CHG–alcohol and PVP-I in alcohol-based solutions.

The concentration of the CHG solution ranges from 0.5 per cent to 4.0 per cent, but there are no data that specify the ideal concentration for surgical preparation to prevent SSI. In contrast, the Centers for Disease Control and Prevention guidelines for the prevention of intravascular catheter-related bloodstream infection recommend preparing clean skin with a higher than 0.5 per cent chlorhexidine (not 0.5 per cent preparation) in alcohol solution. This recommendation is based on the fact that when 0.5 per cent chlorhexidine preparation was compared with 10 per cent PVP-I, no differences were seen in either central venous catheter colonization or catheter-related bloodstream infection^13,14^. Pages *et al.*^15^ reported that compared with PVP-I in alcohol, the incidence of catheter-related infection was lower with 2 per cent chlorhexidine-alcohol and similar with higher than 1 per cent CHG–alcohol after controlling for potential confounders.

A 2 per cent CHG–70 per cent isopropyl alcohol solution has become widely used for both preparation during central venous catheter insertion and preparation of the surgical site^16,17^; however, such compounds are not available in some countries, including Japan, and they often have irritating effects on the skin when a 2 per cent or higher concentration is used^18–20^. The efficacy of a 0.5 per cent or 1.0 per cent chlorhexidine skin preparation with alcohol for the prevention of SSI remains unclear. The present study was performed to identify the concentration of CHG in alcohol-based solution for skin preparation to prevent SSI compared with PVP-I. The secondary aim was to compare the SSI rate between skin preparations of CHG–alcohol and PVP-I in alcohol.

## Methods

### Search strategy

This work is reported according to the PRISMA guidelines^21^. The PRISMA checklist is provided in *Table S1*. Four electronic databases (PubMed, Cochrane Library, Web of Science, and Clinicaltrials.gov) were searched on 5 November 2020. Three reviewers (T.H., S.T., and T.M.) independently searched these literature databases using the following search terms: ‘Chlorhexidine’, ‘Chlorhexidine gluconate’, ‘CHG’, ‘Povidone-iodine’, ‘Povidone-iodines’, ‘Povidone iodines’, ‘Povidone iodine ethanol’, ‘Povidone Iodine’, ‘Povidone-Iodine’, ‘Povidone-Iodines’, ‘PVP-I’, ‘PVP-Iodine’, ‘PVPI’, and 60 other words with one or more search results in the databases (*Table S2*). After the search, the pooled articles were screened and duplicated articles were excluded.

### Selection of studies

Randomized clinical trials (RCTs) that met the following criteria were included in the meta-analysis: comparison of the SSI rate after skin preparations for surgery using CHG–alcohol and PVP-I; use of antiseptics for preparation of the surgical site in the operating room, not for washing (bathing and showering) separately outside of the operating room; and availability of detailed information in English. At least two authors (T.H., T.M., J.K., I.S., Y.T., and T.M.) independently screened the literature. During the screening, disagreements were resolved through discussions with a third reviewer (S.T.).

### Data extraction

Two authors (T.H. and S.T.) independently extracted data from the included studies. Disagreements were resolved by discussions. The following information was extracted: study design, country, study interval, detailed information of each antiseptic (concentration and solution), reported outcome, skin preparations before use of the two antiseptics, observation interval, definition of SSI, patients included, exclusion criteria, number of participants based on intention-to-treat (ITT) (or per-protocol set if ITT was unavailable), number of participants with SSI, and number of participants with adverse events.

### Outcomes analysed

The primary purpose of this study was the efficacy of decreasing the SSI risk by CHG–alcohol *versus* PVP-I (alcohol-based/aqueous solution) according to the CHG concentration. The following terms in each study were defined as SSI: wound infection, postoperative infection, wound complication, and postoperative surgical wound infection. As the secondary purpose of this study, the following analyses for SSI risk were performed: overall comparison between CHG–alcohol and PVP-I; comparison between CHG–alcohol and PVP-I-alcohol; comparison between CHG–alcohol and PVP-I according to the PVP-I concentration; comparison of the two antiseptic groups stratified by wound classification (clean, clean-contaminated, and contaminated wound)^22^; and comparison of the two antiseptic groups stratified by SSI type (superficial incisional, deep incisional, and organ/space SSI)^22^. The comparative risk of adverse events between CHG–alcohol and PVP-I was also evaluated.

### Assessment of risk of bias and publication bias

The two authors (T.H. and S.T.) independently assessed the risk of bias using the Cochrane Handbook for Systematic Reviews of Interventions^23^. The bias assessments performed in the present study were random sequence generation (selection bias); allocation concealment (selection bias); blinding of participants and personnel (performance bias); blinding of outcome assessment (detection bias); incomplete outcome data (attrition bias); selective reporting (reporting bias); and other bias. The risk of other bias was judged to be low when the trials received no financial support from pharmaceutical companies. If sufficient information for assessment was not described, the risk of bias was judged to be unclear. In addition, publication bias was assessed by visual examination of a funnel plot and statistical analyses using Egger’s test.

### Results analyses and statistical analyses

The extracted data were analysed using Review Manager for Windows (RevMan version 5.4.1; The Nordic Cochrane Centre, The Cochrane Collaboration, Copenhagen, Denmark), and forest plots were prepared. The Mantel–Haenszel random-effects model was used to calculate the risk ratios (RRs) and 95 per cent confidence intervals (95 per cent c.i.). Between-study heterogeneity was quantified using the *I*^2^ statistic, which was assessed according to the following criteria: *I*^2^ less than 25 per cent, no heterogeneity; *I*^2^ of 25–50 per cent, moderate heterogeneity; and *I*^2^ greater than 50 per cent, high heterogeneity. A *P* value of less than 0.050 was considered to indicate a significant difference.

## Results

### Literature search results

**Fig. 1:**
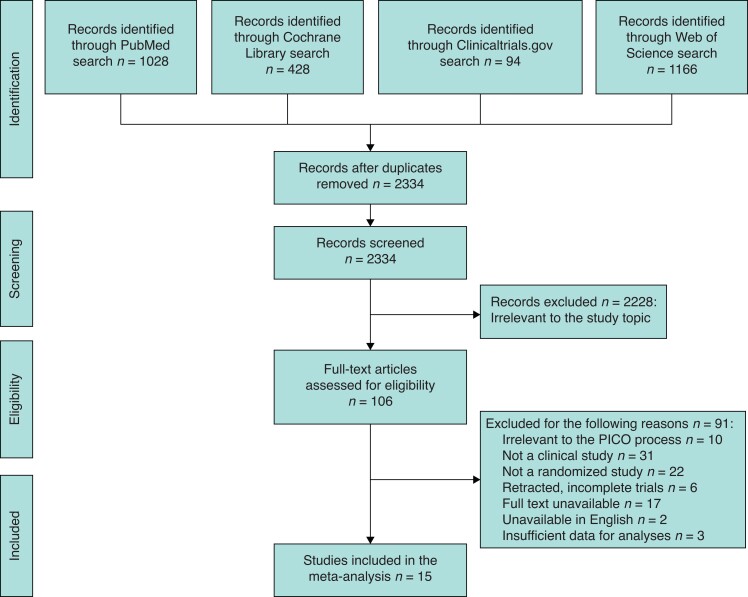
Flowchart of study selection PICO, Population, Intervention, Comparison, Outcome.

*Figure 1* shows the screening and selection of studies. From the four electronic databases, 2716 articles were obtained to be screened and 382 duplicate articles were excluded. After screening the titles and abstracts, 2228 articles were excluded, and 106 articles were retrieved for full-text review. Of these 106 articles, 91 did not meet the inclusion criteria. Finally, 15 studies^24–38^ were included in the meta-analysis.

### Characteristics of included studies and participants and assessment of risk of bias

**Fig. 2:**
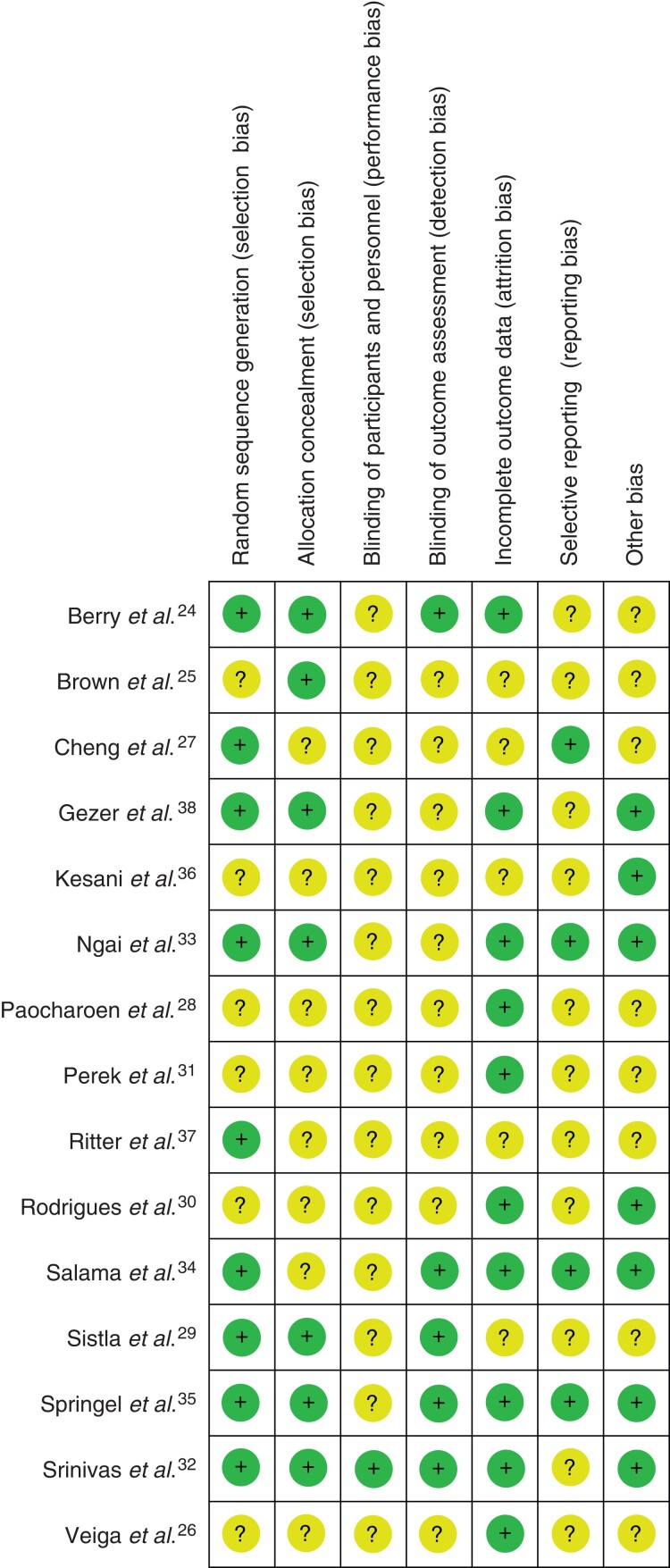
Summary of risk-of-bias assessment

*Table 1* summarizes the characteristics of the studies included in the present meta-analysis. The CHG concentration ranged from 0.5 per cent to 4.0 per cent, and the PVP-I concentration ranged from 1.0 per cent to 10.0 per cent. Data on the CHG and PVP-I concentrations were unavailable in two studies^31,33^ and four studies^28,31,33,38^ respectively. Of 15 included studies, 14 studies described the follow-up interval, and nine studies had a follow-up of 30 days. *Table 2* summarizes the characteristics of the participants. In each article, the number of participants was extracted based on ITT. The number of ITT participants was unavailable in three studies^25,30,37^; therefore, the number of per-protocol set patients was extracted instead of ITT participants in these three studies. In total, 6974 participants were involved in the studies: 3472 participants were disinfected with CHG–alcohol and 3502 participants were disinfected with PVP-I. *Figure 2* indicates the risk of bias for each study. No studies were judged to have a high risk of bias among all included studies. The blinding of participants and personnel (performance bias) was unclear in all included studies except for Srinivas *et al*^32^.

**Table 1:** Characteristics of studies included in the present meta-analysis

Study	Study design	Country, time interval	CHG (concentration, solution) (%)	PVP-I (concentration, solution) (%)	Reported outcome	Skin washing before use of the two antiseptics		Observation interval	Definition of SSI
						CHG	PVP-I		
Berry 1982^24^	Prospective, randomized study	England, 1978–1980	0.5, 70 isopropyl alcohol	A: 10, alcohol B: 7.5, N/A	Wound infection	Two applications with sterile sponges		3 to 4 days	N/A
Brown 1984^25^	Prospectively randomized study	India, 1979–1980	0.5, 70 isopropyl alcohol	7.5, N/A	Wound infection	Removal of obvious foreign material present with a clean sponge followed by a spray application of 0.5% CHG in 70% isopropyl alcohol	6 min scrub with PVP-I soap, then painted with aqueous PVP-I solution that absorbed with a sterile towel	N/A	A minor wound infection: an infected wound with superficial, separation (less than 1 cm) involving less than one-third of the incision or induration of the wound edge believed by the surgeon to be secondary to infection. A major wound infection: an infected wound with separation of the wound edges greater than one-third of the length of the incision or frank wound infection with evidence of purulent exudate or abscess.
Veiga 2008^26^	RCT	Brazil, N/A	0.5, alcohol	10, alcohol	Postoperative infection	A vigorous scrub with antiseptic soap, followed by absorption with a sterile towel and painting		30 days	The CDC definitions and classification of surgical site infections
Cheng 2009^27^	Prospective randomized study	England, 2007–2008	0.5, 70 isopropyl alcohol	10, 23 isopropyl alcohol	Postoperative infections or wound complications	Scrubbed with a sterile surgical bristled brush for 3 min and then painted		N/A	N/A
Paocharoen 2009^28^	Prospective randomized trial	Thailand, 2006–2008	4, 70 isopropyl alcohol	N/A, N/A	Postoperative surgical wound infection	5 min scrubbing, then paint		1 month	If a surgical wound drained purulent material or if the surgeon judges it to be infected and opens it
Sistla 2010^29^	Prospective randomized trial	India, N/A	2.5, 70 ethanol	10, N/A	SSI	Applied in concentric circles beginning from the site of incision to the periphery and allowed to dry before the surgical site was draped		30 days	The CDC criteria
Rodrigues 2013^30^	Randomized, longitudinal study	Brazil, 2011	0.5, alcohol	10, hydro alcohol	SSI	The skin was prepared in the same manner as it was for the PVP-I group; however, the cleaning was carried out with water and 20 ml 2% CHG soap, and complementation with 0.5% alcoholic CHG	After hand hygiene and gloving, with a compress soaked in water and 20 ml PVP-I, the area was vigorously rubbed for 5 min. The area was then cleaned with another sterile compress. The preparation was completed by marking the operative area with 10% hydroalcoholic PVP-I	N/A	The presence of at least one of the following signs: fever, without other apparent cause, pain, heat, swelling, or confluent erythema around the incision and extrapolating the boundaries of the wound, pus in the incision site or in the deep soft tissue, or in organ/cavity handled during operation; presence of abscesses or, in the case of deep tissues, histological or radiological evidence suggestive of infection; isolated microorganism from theoretically sterile source or harvested with aseptic technique from a previously closed site, and spontaneous dehiscence of deep tissues
Perek 2013^31^	Randomized clinical study	Poland, 2011	N/A, 70 ethanol	N/A, 50 propyl alcohol	SSI	Had a shower and a bath with CHG soap on the day before surgery, then disinfected twice		30 days	CDC guidelines
Ngai 2015^33^	RCT	USA, 2013–2014	N/A, alcohol	N/A, alcohol	SSI	CHG with alcohol	PVP-I with alcohol	30 days	According to Horan *et al.* 1992 and the CDC
Srinivas 2015^32^	RCT	India, 2011–2012	0.5, 70 isopropyl alcohol	5, N/A	SSI	Painted 3 times, around the site of the incision	Painted with 5% PVI-I solution three times	30 days	CDC criteria
Salama 2016^34^	RCT	Egypt, 2014	2, 70 alcohol	10, 70 alcohol	SSI	3 applications of 2% CHG followed by drying with a sterile towel after 30 and 3 applications of 70% alcohol	Scrubbed that contained 10% PVP-I, followed by drying with a sterile towel after 1 min and 3 applications of 10% PVP-I in 70% alcohol	30 days	Defined by pain, tenderness, swelling, redness, heat, purulent discharge from the incision, or deliberate reopening of the surgical wound
Springel 2017^35^	RCT	USA, 2014–2016	2, 70 isopropyl alcohol	10, aqueous	SSI	Paint	0.75% PVP-I aqueous scrub followed by 1.0% PVP-I aqueous paint	N/A	US National Healthcare Safety Network, CDC definitions
Kesani 2019^36^	Randomized prospective study	N/A, 2017	2, 70 isopropyl alcohol	10, surgical spirit	SSI	Before operation, scrubbed at the surgical site by either the CHG–alcohol (2% CHG and 70% isopropyl alcohol)	Before operation, scrubbed at the surgical site by either the PVP-I (10% PVP-I and then with surgical spirit)	30 days	CDC definitions
Ritter 2019^37^	Prospective randomized trial	N/A, 2014–2015	2, 70 isopropyl alcohol	1, 50 2-propanol	SSI	ChloraPrep (2% CHG and 70% isopropyl alcohol) (CareFusion; Leawood, Kansas, USA)	Braunoderm (1% PVP-I and 50% 2-propanol) (B. Braun Medical AG; Melsungen, Germany)	6 months	Established criteria published by the CDC and the following additional criteria: (1) necessity of antibiotic therapy, (2) necessity of surgical intervention, and (3) positive microbiologic culture of swabs taken intraoperatively
Gezer 2020^38^	RCT	Turkey, 2017–2019	4, alcohol	N/A, N/A	SSI	Habitanol 1000 ml solution (Kimpa Drugs, İstanbul, Turkey)	Poviderm 1000 ml solution (Necm Chemistry, İstanbul, Turkey)	30 days	CDC definition

N/A, not available; CHG, chlorhexidine gluconate; PVP-I, povidone-iodine; RCT, randomized clinical trial; SSI, surgical site infection; CDC, Centers for Disease Control and Prevention.

**Table 2:** Characteristics of participants

Study	Patients included	Exclusion criteria	Number of participants		Number of SSIs	
			CHG–alcohol	PVP-I	CHG–alcohol	PVP-I
Berry 1982^24^	Elective surgical cases	Patients sensitive to one or other preparation.	453	413	44	61
Berry 1984^25^	Patients from both private and clinic services	Patients underwent operations not included in the study protocol. Patients with death within 48 h of the operation. Patients required a second operation within 48 h.	378*	359*	23	29
Veiga 2008^26^	Age 18 years or older Scheduled for elective and clean plastic surgery procedures	N/A	0	125	4	125
Cheng 2009^27^	Undergoing foot surgery	Patients with current open wounds skin ulcers and/or sores. Patients with a history of onychomycosis, paronychia, or nail deformity. Patients with poorly controlled diabetes mellitus or recent antibiotic use (within 1 week of surgery).	0	25	0	25
Paocharoen 2009^28^	Age 18–60 years	Patient refusal, dirty wound, uncontrolled diabetes, on immunosuppressive drugs, serum albumin less than 3.0 mg/dl. Patients with a history of allergy to study agent.	250	250	5	8
Sistla 2010^29^	Elective inguinal hernia repair	Patients with recurrent or complicated inguinal hernia. Patients with a history of allergy to the antiseptics.	271	285	14	19
Rodrigues 2013^30^	Age 18 years or older Open-access elective procedures, with subcostal abdominal, vertical abdominal and thoracic incisions	Patients with breaches in the rules of antisepsis and asepsis, changing the classification of the surgical site. Patients with abandoned follow-up.	103*	102*	11	7
Perek 2013^31^	Elective cardiac procedures carried out via median sternotomy	Patients with pre-existing infections (for example infective endocarditis) treated with antibiotics. Patients operated on emergently due to complications resulting from percutaneous interventions. Patients treated surgically for aortic aneurysms or acute dissections (due to more aggressive perioperative antibiotic prophylaxis). Patients requiring prolonged (exceeding 72 h) intubation and mechanical ventilation.	47	47	2	4
Ngai 2015^33^	Gestation period 37 or more weeks on best obstetric estimate scheduled or non-emergent Caesarean delivery	Patients had a urogenital tract infection within 2 weeks of delivery. Patients with a 2-week or more history of steroid delivery during their pregnancy. Patients younger than 18 years old.	474	463	21	21
Srinivas 2015^32^	Age 18–70 years Uniformly received the preoperative antibiotic during the induction of anaesthesia	Patients with no consent for the trial. Patients with a history of allergy to CHG, alcohol, or iodophors. Clinical/microbiological evidence of infection at/adjacent to the surgical site. Patients with ongoing systemic sepsis. Patients died intraoperatively or before the completion of the 30-day follow-up interval. Patients left the hospital against medical advice or lost to follow-up. Patients required a second operation within two weeks of the first operation.	163	188	17	33
Salama 2016^34^	Age 18–42 years BMI 20–35 Elective and non-elective Caesarean sections	Patients with a history of allergy to CHG, alcohol, and iodophors. Patients with a history of rupture of membranes more than 24 h Patients with documented concomitant infections such as chorioamnionitis, pyelonephritis, mastitis. Patients with diabetics or obese. Patients with BMI greater than 35.	204	201	9	27
Springel 2017^35^	Age 18 years or older Delivery, or intrapartum once a plan for Caesarean	Patients with no key study personnel to complete study-related procedures. Patients allergic to PVP-I or CHG. Patients with diagnosed with clinical chorioamnionitis. Patients incarcerated. Study personnel perceived that the patient was unlikely to return to complete postoperative assessments. Patients unable or unwilling to consent for study participation in English or Spanish.	461	471	29	33
Kesani 2019^36^	Age 18 years or older Caesarean sections	Patients with a history of allergy to CHG, alcohol, or iodophors evidence of infection at or adjacent to the operative site. Patients with no follow-up the patient’s course for 30 days after surgery.	296	296	19	41
Ritter 2019^37^	Elective or emergency traumatological surgery of the lower leg, ankle, or foot at a single institution	Patients with history of systemic disease (for example dermatitis herpetiformis, or Duhring’s disease). Patients with an allergy to the researched agents or one of its components. Underage participants (under 18 years old). Polytraumatized participants. Participants with open fractures or manifest infections.	112*	167*	2	9
Gezer 2020^38^	Surgery for malignant or premalignant conditions of the uterus, cervix or ovary, or peritoneal carcinomatosis	Patients unable to give informed consent. Patients with a known allergy to the disinfectants. Patients currently using antimicrobials, immunosuppressant drugs, or insulin for uncontrolled diabetes. Patients with an open wound.	110	110	12	12

N/A, not available; CHG, chlorhexidine gluconate; PVP-I, povidone-iodine; SSI, surgical site infection. * Extraction of data based on per-protocol set.

### Overall comparison of SSI rate between CHG–alcohol and PVP-I

**Fig. 3:**
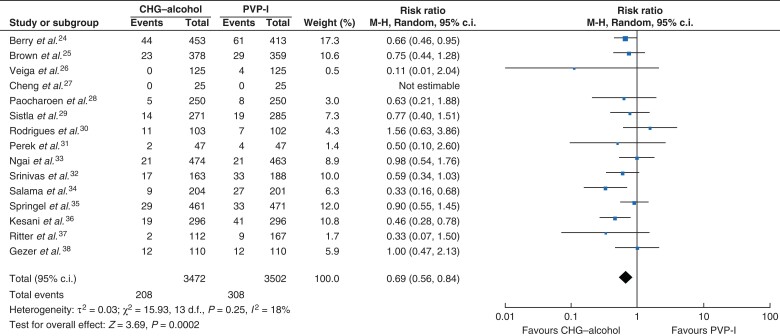
Forest plot comparing SSI rate between CHG–alcohol and PVP-I Risk ratios are represented by diamond shapes, and 95 per cent confidence intervals (c.i.) are represented by horizontal lines. CHG, chlorhexidine gluconate; PVP-I, povidone-iodine.

SSIs were detected in 516 participants (208 in the CHG–alcohol group and 308 in the PVP-I group). CHG–alcohol was significantly more effective than PVP-I (RR = 0.69, 95 per cent c.i. 0.56 to 0.84, *P* = 0.0002, *I*^2^ = 18 per cent) (*Fig. 3*).

### Comparison of SSI rate between CHG–alcohol and PVP-I (alcohol-based/aqueous solution) according to CHG concentration

**Fig. 4:**
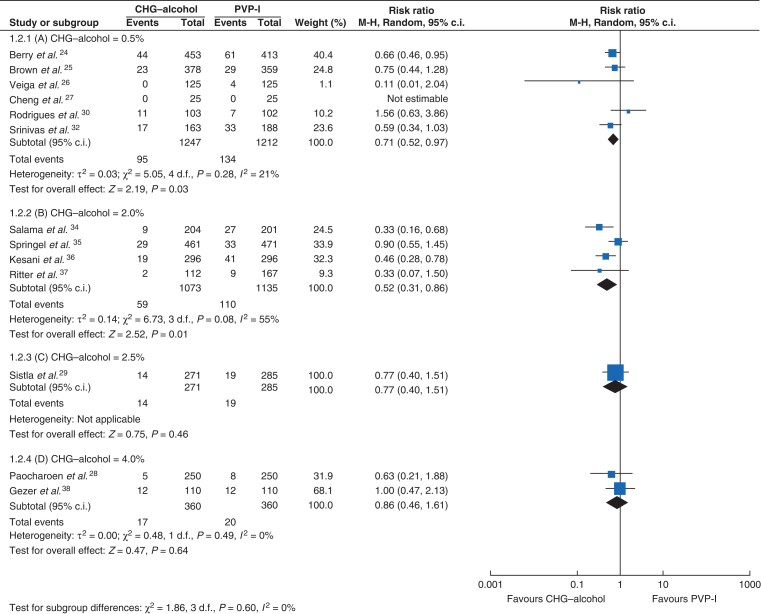
Forest plot of subgroup analyses comparing SSI rate among different concentrations ranging from 0.5 per cent to 4.0 per cent CHG–alcohol and PVP-I. (a) CHG–alcohol = 0.5%, (b) CHG–alcohol = 2.0%, (c) CHG–alcohol = 2.5%, (d) CHG–alcohol = 4.0% Risk ratios are represented by diamond shapes, and 95 per cent confidence intervals (c.i.) are represented by horizontal lines. CHG, chlorhexidine gluconate; PVP-I, povidone-iodine.

The CHG concentrations in CHG–alcohol were stratified into 0.5 per cent, 2.0 per cent, 2.5 per cent, and 4.0 per cent. No RCTs compared 1.0 per cent CHG–alcohol and PVP-I. Six studies compared 0.5 per cent CHG–alcohol and PVP-I^24–27,30,32^. The SSI rate in the 0.5 per cent CHG–alcohol group was significantly lower than that in the PVP-I group (RR = 0.71, 95 per cent c.i. 0.52 to 0.97, P = 0.03, *I*^2^ = 21 per cent) (*Fig. 4a*). Four RCTs compared 2.0 per cent CHG–alcohol and PVP-I^34–37^. A significantly lower SSI rate was observed in the 2.0 per cent CHG–alcohol group than in the PVP-I group (RR = 0.52, 95 per cent c.i. 0.31 to 0.86, P = 0.01, *I*^2^ = 55 per cent) (*Fig. 4b*). No significant difference in the SSI rate was found between the greater than 2.0 per cent CHG group and the PVP-I group (2.5 per cent CHG, RR = 0.77, 95 per cent c.i. 0.40 to 1.51, P = 0.46; 4.0 per cent CHG, RR = 0.86, 95 per cent c.i. 0.46 to 1.61, P = 0.64, *I*^2^ = 0 per cent) (*Fig. 4c,d*), possibly because of the lack of power caused by an insufficient number of included studies. Other PVP-I concentrations, commonly using concentrations such as 5 per cent and 7.5 per cent, could not be analysed because only one study was available at each concentration.

### Other comparisons of SSI rate between CHG–alcohol and PVP-I

**Fig. 5:**
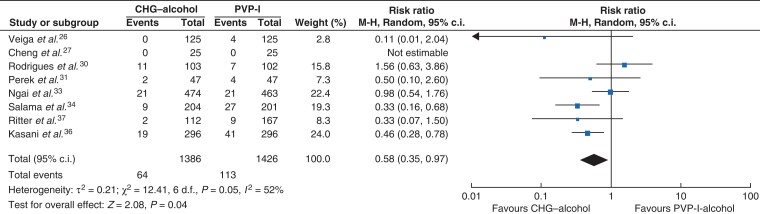
Forest plot comparing SSI rate between CHG–alcohol and PVP-I-alcohol Risk ratios are represented by diamond shapes, and 95 per cent confidence intervals (c.i.) are represented by horizontal lines. CHG, chlorhexidine gluconate; PVP-I, povidone-iodine.

In the comparison among the alcohol-based solutions (*Fig. 5*), CHG–alcohol was associated with a significantly lower SSI rate than alcohol-based PVP-I (RR = 0.58, 95 per cent c.i. 0.35 to 0.97, P = 0.04, *I*^2^ = 52 per cent)^26,27,30,31,33,34,36,37^. In the comparison of the SSI risk associated with these antiseptic solutions according to the PVP-I concentration (lower than 10 per cent and 10 per cent), significantly higher risks were found with PVP-I irrespective of the PVP-I concentration (RR = 0.67, 95 per cent c.i. 0.46 to 0.98, *P* = 0.04, *I*^2^ = 0 per cent and RR = 0.62, 95 per cent c.i. 0.40 to 0.96, *P* = 0.03, *I*^2^ = 53 per cent respectively) (*Fig. S1a*,*b*).

Subgroup analyses of the effectiveness of preventing SSI between CHG–alcohol and PVP-I were conducted according to the wound classification (*Fig. S2a–c*) and SSI type (*Fig. S3a–c*). Four studies showed data for clean wounds, four for clean-contaminated wounds, and one for contaminated wounds. The effectiveness of CHG–alcohol over PVP-I was demonstrated only for clean-contaminated wounds (RR = 0.56, 95 per cent c.i. 0.37 to 0.85, *P* = 0.006, *I*^2^ = 50 per cent) (*Fig. S2b*); no significant difference was observed in the other wound classes (*Fig. S2a*,*c*). In the analyses of SSI type, significant benefits in reducing the SSI risk with CHG–alcohol compared with PVP-I were observed for superficial incisional SSI^26,30–38^ and deep incisional SSI^30–37^ (RR = 0.71, 95 per cent c.i. 0.54 to 0.93, *P* = 0.01, *I*^2^ = 9 per cent and RR = 0.47, 95 per cent c.i 0.24 to 0.91, *P* = 0.03, *I*^2^ = 0 per cent respectively) (*Fig. S3a*,*b*). No significant difference between the two antiseptic groups was observed for organ/space SSI (RR = 1.23, 95 per cent c.i. 0.54 to 2.82, *P* = 0.62, *I*^2^ = 0 per cent)^30,32–35,38^ (*Fig. S3c*).

### Comparison of adverse events between CHG–alcohol and PVP-I

Allergic reactions were the only reported adverse events in the included studies, and no significant difference was observed between the CHG–alcohol group and PVP-I group (RR = 0.75, 95 per cent c.i. 0.17 to 3.29, *P* = 0.70) (*Fig. S4*)^32,34,36^.

### Assessment of publication bias

**Fig. 6:**
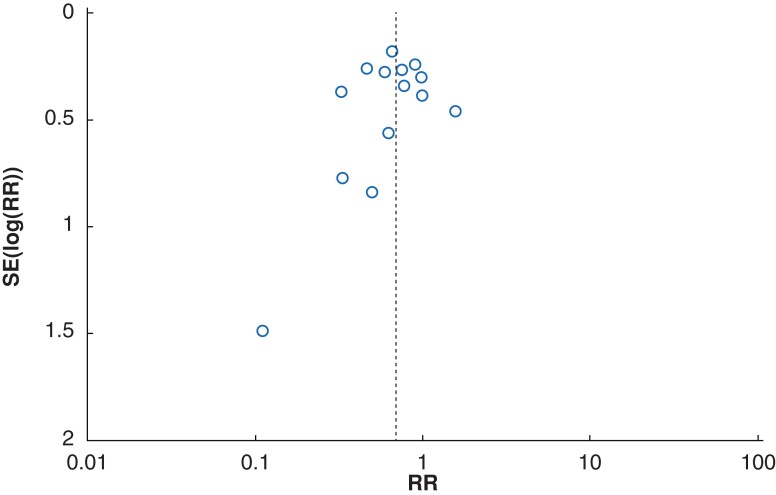
Funnel plot of primary outcome SSI rate based on 15 studies comparing chlorhexidine gluconate-alcohol and povidone-iodine. The dashed line indicates the pooled risk ratio (RR) of 0.69.

Publication bias was assessed using funnel plotting and Egger’s test (*Fig. 6*). No statistically significant publication bias was found (*P* = 0.5703).

## Discussion

This is the first meta-analysis to compare the risk of SSI between CHG–alcohol and PVP-I according to the CHG concentration. The results showed that both 2.0 per cent compound and 0.5 per cent compound more effectively prevented SSI than PVP-I. This is an important step in clinical practice for countries in which 2 per cent CHG–alcohol is not available. Because of the risk of anaphylaxis, Japanese pharmaceutical regulations prohibit the application of CHG to mucosal surfaces, including in dental care, and limit the CHG concentration in skin antiseptics to a maximum of 1 per cent.

To determine the recommended CHG concentration in CHG–alcohol, direct comparison with different CHG concentrations is required. In one study, the antimicrobial activity of a 2.0 per cent CHG–alcohol solution was superior to that of a 0.5 per cent CHG–alcohol solution when challenged with a *Staphylococcus epidermidis* biofilm^39^; however, significantly increased preventative effects against SSI have not been demonstrated by head-to-head RCTs between different CHG concentrations. Three studies compared the efficacy of CHG–alcohol with different CHG concentrations by skin cultures. In a study that evaluated the mean bacterial count reductions for the use of surgical skin preparation, the antimicrobial effectiveness of 1.0 per cent CHG–alcohol was superior to that of 0.5 per cent CHG–alcohol, particularly at the abdominal site^40^. CHG–alcohol has immediate and persistent activity, with the alcohol having a rapid mode of action and the CHG offering residual activity. CHG binds to anionic cutaneous protein, resulting in a prolonged antiseptic effect. Hence, a higher concentration might be required for surgical skin preparation.

Similarly, Casey *et al.*^41^ compared 0.5 per cent CHG–alcohol with 2.0 per cent CHG–alcohol for skin antisepsis in patients undergoing vein graft harvesting for coronary artery bypass graft surgery. There was a significant difference in the culture-positive rate between 0.5 per cent CHG–alcohol and 2.0 per cent CHG–alcohol after incision closure, which occurred at approximately 90 min after application of the skin antiseptics in each group (33.3 *versus* 12.5 per cent respectively). In addition, significantly fewer microorganisms within the adhesive dressings removed 24 h after application were observed in the 2.0 per cent CHG–alcohol group than in the 0.5 per cent CHG–alcohol group, which might indicate that 2.0 per cent CHG–alcohol more effectively kills microorganisms located in the lower layers of the skin. In contrast, Nishihara *et al.*^42^ reported that there was no significant difference in the log reduction of the bacterial count among CHG preparations of 0.5 per cent, 1.0 per cent, and 2.0 per cent.

Although the precise prevalence of CHG allergy is unclear, the numbers of case reports describing such allergy have recently increased, especially in the perioperative setting^43–47^. High concentrations of CHG (2–4 per cent) possibly have irritant effects on the skin, leading to an impaired skin barrier and increasing the risk of allergy^18,43,48^. Nishihara *et al.*^40^ reported the mean visual scores of skin irritation and the total cumulative irritation scores after repeated exposure to test products, and lower scores were found in the 1 per cent than 2 per cent CHG–alcohol group. The potential risks *versus* benefits should be considered before proposing an adequate CHG concentration.

Previous meta-analyses have shown that alcohol-based antiseptic solutions are more effective than aqueous solutions in reducing the risk of SSI^6^. The present meta-analysis demonstrated that CHG was more protective than PVP-I in the evaluation limited to alcohol-based solution. Skin preparation is performed to prevent wound infection, and this meta-analysis confirmed that CHG–alcohol was significantly more protective than PVP-I against both superficial and deep incisional SSI but not against organ-space SSI. Surgical skin preparation with CHG–alcohol was superior to skin preparation with PVP-I for preventing SSI only after clean-contaminated surgery. An additional RCT is required to evaluate the effectiveness of CHG–alcohol in clean surgery.

This study had some limitations. First, washing the patient’s skin with antiseptics, which was performed separately outside the operating room, might have impacted the results. Some studies adopted the same antiseptic compound for both preoperative body washing (CHG soap or PVP-I soap) and skin preparation at the surgical site. Second, bias caused by the heterogeneity of SSI prevention protocols, including antimicrobial prophylaxis, and normothermia, should be considered. Of the 15 studies, 12 described the use of prophylactic antimicrobials; however, it was not possible to compile detailed information on criteria for use. Third, comparison with PVP-I in alcohol should be performed to confirm the effectiveness of 0.5 per cent CHG–alcohol. Fourth, considering the time course between the first^24^ and the last^38^ studies included in this meta-analysis, the improvement of medical care and medical technology during this interval should be considered as an important confounder. Last, only three studies^26,33,34^ described the time of exposure. Although application time of 3–5 min is recommended in PVP-I solution, a shorter drying time is permitted in CHG–alcohol^49,50^.

An alcohol-based CHG solution with a CHG concentration of 0.5 per cent or higher can be used for surgical skin preparation to prevent SSI. CHG–alcohol was more effective than PVP-I irrespective of the type of solution (alcohol *versus* aqueous). Additional studies are required to propose an adequate CHG concentration by head-to-head comparison of the SSI rate and skin complications according to the CHG concentration.

## Supplementary Material

zrac111_Supplementary_Data

## Data Availability

All data generated or analysed during this study are included in this published article.
