# Supplementary material for: Efficacy of surgical skin preparation with chlorhexidine in alcohol according to the concentration required to prevent surgical site infection: meta-analysis
Source: BJS Open. 2022 Sep 19;6(5):zrac111. doi: 10.1093/bjsopen/zrac111 (PMC9487656; doi:10.1093/bjsopen/zrac111)
Supplement: zrac111_Supplementary_Data [file zrac111_supplementary_data.zip › Supplementary_Table_2.docx]

Table S2. Search term and formula.

| # | Search Term |
| --- | --- |
| 1 | Adle |
| 2 | Aerodine |
| 3 | Alphadine |
| 4 | Ashru |
| 5 | Betadine |
| 6 | Betadine Surgical Scrub |
| 7 | Betagen |
| 8 | Betaisodona |
| 9 | Biozide |
| 10 | Clinidine |
| 11 | Dermawound |
| 12 | E-Z Scrub Povidone Iodine |
| 13 | ExCel AP |
| 14 | Front Eye |
| 15 | Healin |
| 16 | iodopovidone |
| 17 | Isodine |
| 18 | Loris |
| 19 | Mallisol |
| 20 | Moist |
| 21 | Pentadine |
| 22 | Petrom |
| 23 | Polydine |
| 24 | Povidex |
| 25 | Povidine |
| 26 | Prevail |
| 27 | Prevail-FX |
| 28 | Pyodine |
| 29 | Rexall |
| 30 | Triadine |
| 31 | Povidone - iodine |
| 32 | Povidone - iodines |
| 33 | Povidone Iodine |
| 34 | Povidone iodine ethanol |
| 35 | Povidone iodines |
| 36 | Povidone-Iodine |
| 37 | Povidone-Iodines |
| 38 | PVP-I |
| 39 | PVP-Iodine |
| 40 | PVPI |
| 41 | #1 OR #2 OR #3 OR ... OR #40 |
| 42 | Avagard |
| 43 | Bactoshield |
| 44 | Calgon Vesta |
| 45 | CHG SCRUB |
| 46 | ChloraPrep |
| 47 | ChloraPrep One-Step |
| 48 | Chlorostat |
| 49 | EXIDINE |
| 50 | Gibitan |
| 51 | Hibiclen |
| 52 | Hibident |
| 53 | Hibidil |
| 54 | Hibisoft |
| 55 | Hibisol |
| 56 | Hibistat Towelette |
| 57 | Hibitane |
| 58 | MASKIN |
| 59 | Micro shield |
| 60 | MICROSHIELD |
| 61 | Muskin |
| 62 | Prevantics |
| 63 | PREVANTICS MAXI SWABSTICK |
| 64 | PREVANTICS SWAB |
| 65 | PREVANTICS SWABSTICK |
| 66 | READYPREP CHG |
| 67 | Scrub Care Exidine |
| 68 | SOLUPREP |
| 69 | Spectrum-4 |
| 70 | STERICLON |
| 71 | Super 2X |
| 72 | Chlorhexidine |
| 73 | Chlorhexidine gluconate |
| 74 | CHG |
| 75 | #42 OR #43 OR #44... OR #74 |
| **76** | **#41 AND #75** |
