# Supplementary figures and images for: Efficacy of surgical skin preparation with chlorhexidine in alcohol according to the concentration required to prevent surgical site infection: meta-analysis
Source: BJS Open. 2022 Sep 19;6(5):zrac111. doi: 10.1093/bjsopen/zrac111 (PMC9487656; doi:10.1093/bjsopen/zrac111)

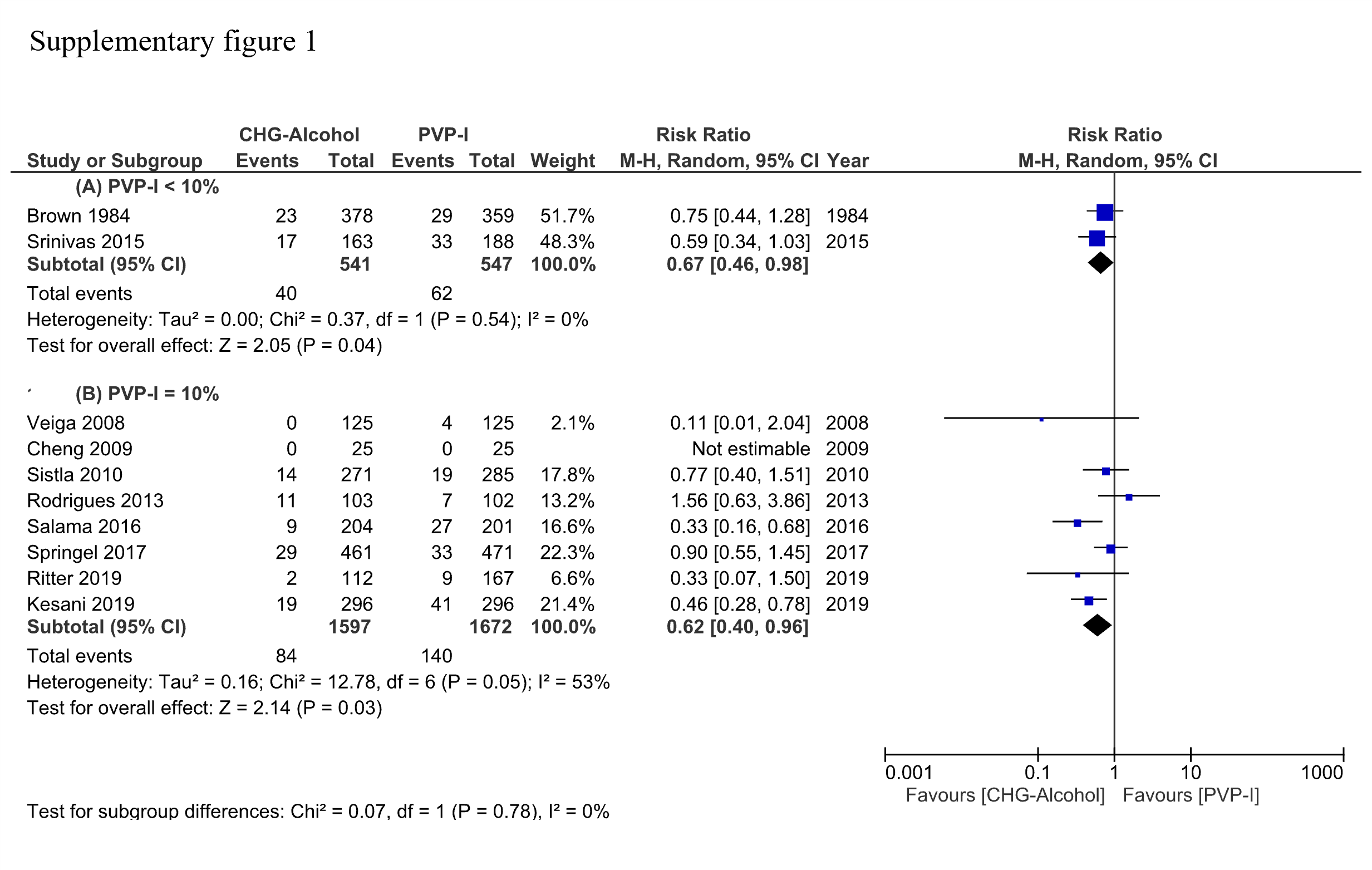

Supplement: zrac111_Supplementary_Data [file zrac111_supplementary_data.zip › Supplementary_Figure_1.tif]

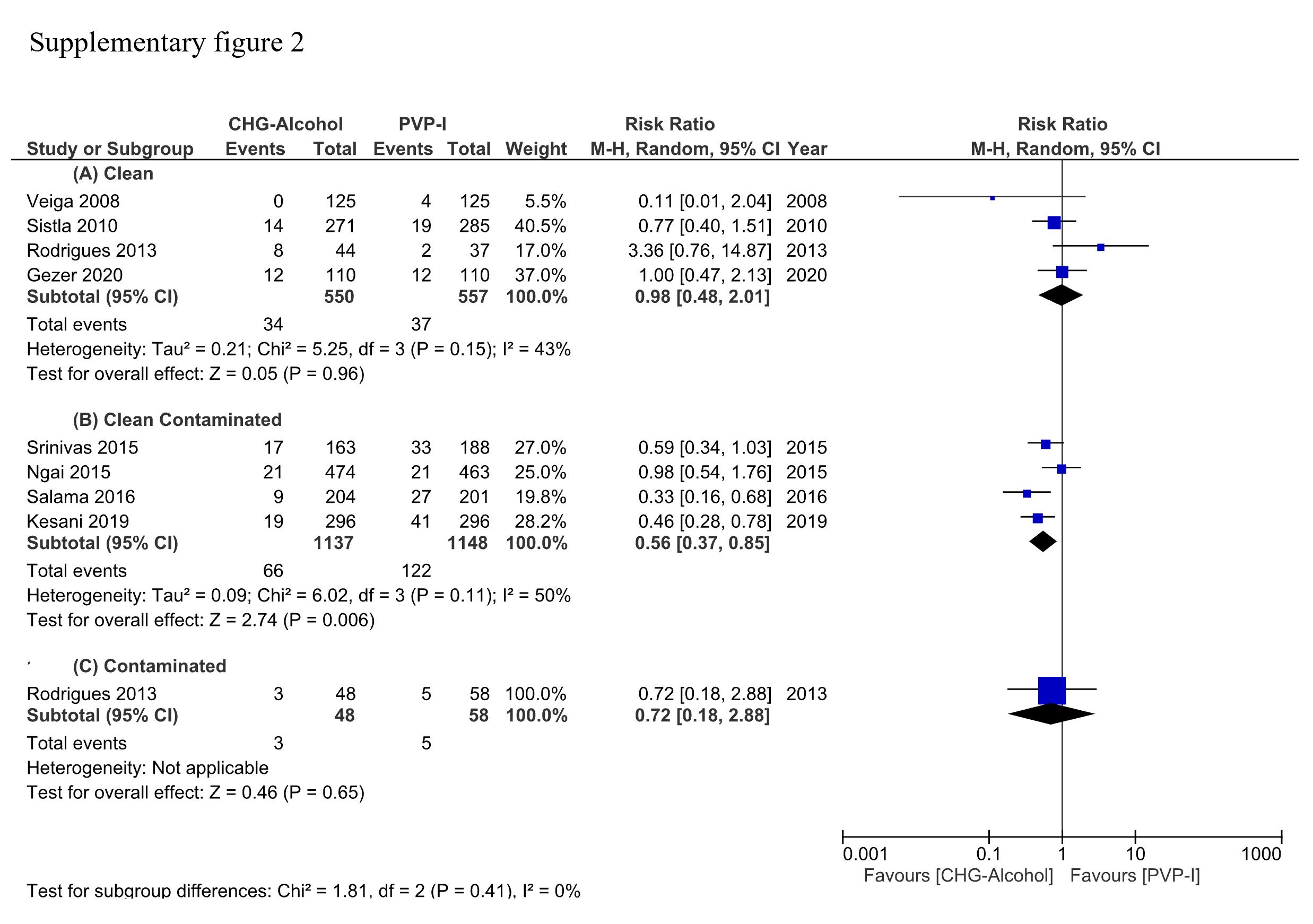

Supplement: zrac111_Supplementary_Data [file zrac111_supplementary_data.zip › Supplementary_Figure_2.tif]

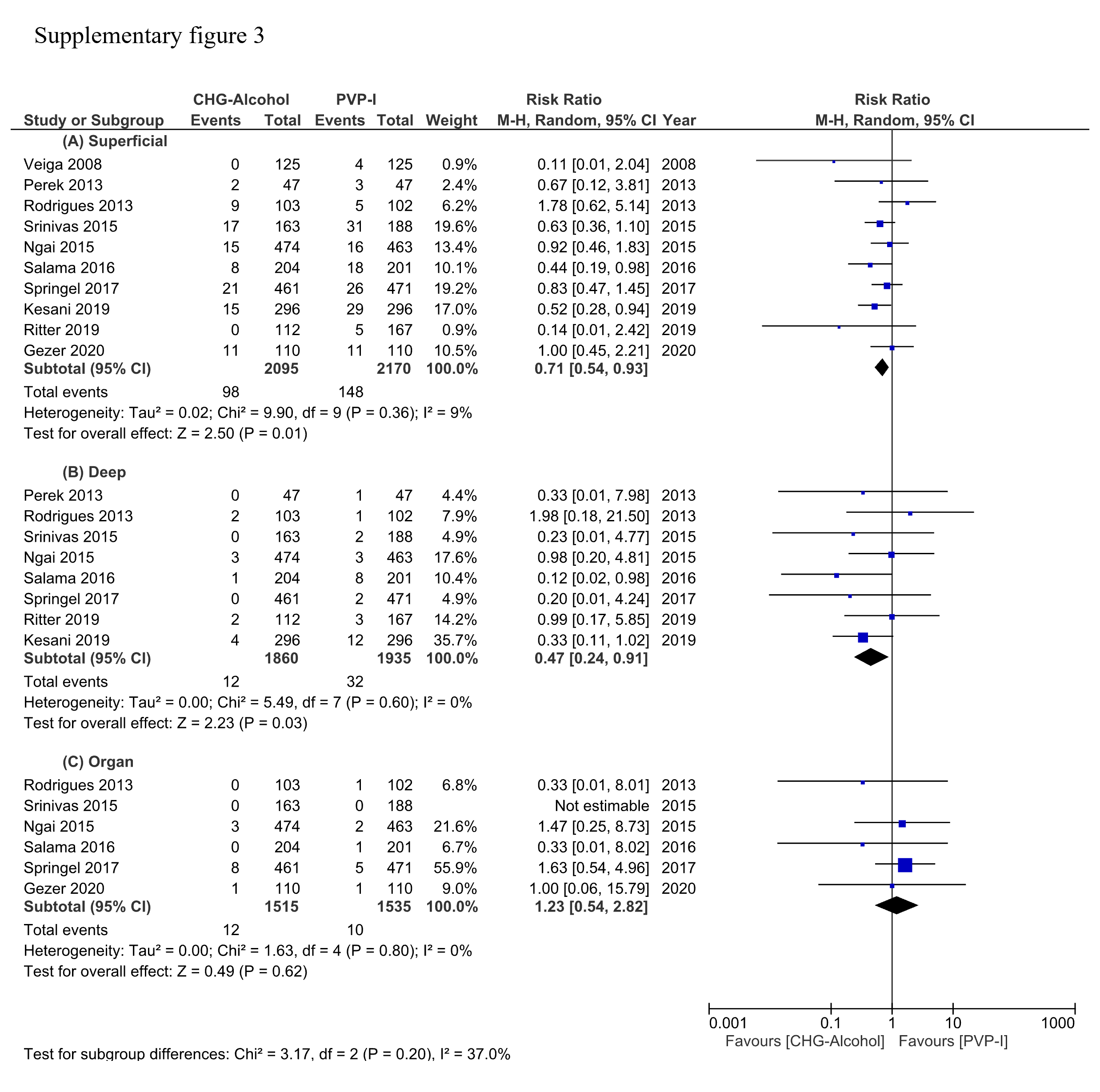

Supplement: zrac111_Supplementary_Data [file zrac111_supplementary_data.zip › Supplementary_Figure_3.tif]

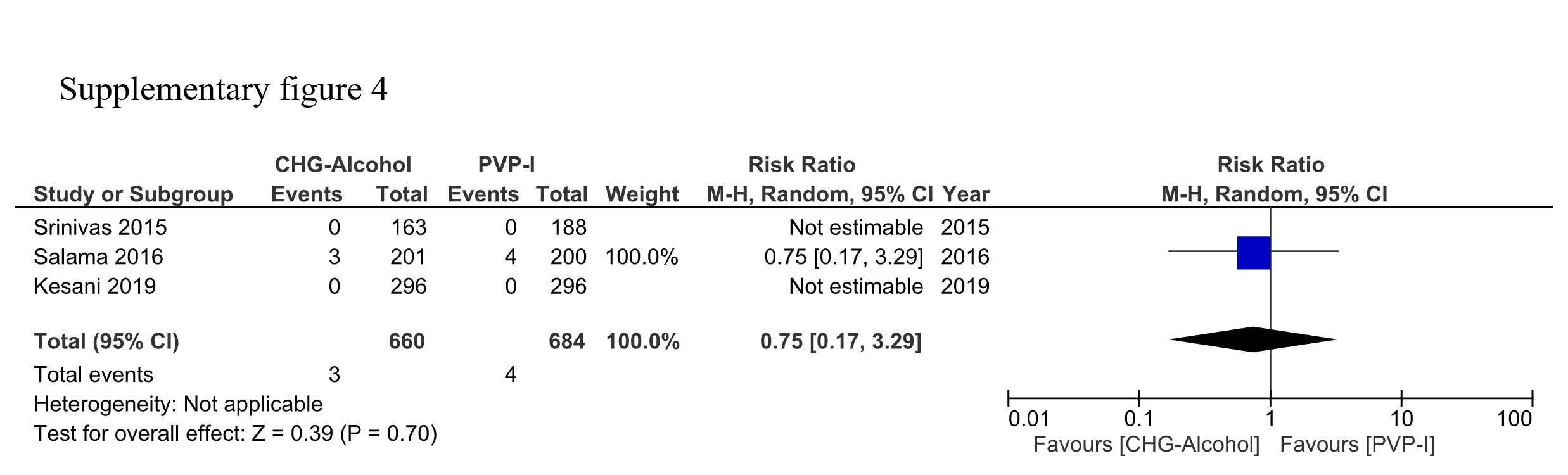

Supplement: zrac111_Supplementary_Data [file zrac111_supplementary_data.zip › Supplementary_Figure_4.tif]
